# Aberrantly Glycosylated GLUT1 as a Poor Prognosis Marker in Aggressive Bladder Cancer

**DOI:** 10.3390/ijms25063462

**Published:** 2024-03-19

**Authors:** Eduardo Ferreira, Dylan Ferreira, Marta Relvas-Santos, Rui Freitas, Janine Soares, Rita Azevedo, Luís Pedro Afonso, Luís Lima, Beatriz Santos, Martina Gonçalves, André M. N. Silva, Lúcio Lara Santos, Andreia Peixoto, José Alexandre Ferreira

**Affiliations:** 1 Experimental Pathology and Therapeutics Group, Research Center of IPO-Porto (CI-IPOP), 4200-072 Porto, Portugal; i37310@ipoporto.min-saude.pt (E.F.); i38795@ipoporto.min-saude.pt (D.F.); marta.relvas.santos@ipoporto.min-saude.pt (M.R.-S.); rui.freitas@ipoporto.min-saude.pt (R.F.); janinepaivasoares@gmail.com (J.S.); luis.afonso@ipoporto.min-saude.pt (L.P.A.); luis.carlos.lima@ipoporto.min-saude.pt (L.L.); beatriz.santos@ipoporto.min-saude.pt (B.S.); martina.goncalves@ipoporto.min-saude.pt (M.G.); lucio.santos@ipoporto.min-saude.pt (L.L.S.); 2 RISE@CI-IPOP (Health Research Network), Portuguese Oncology Institute of Porto (IPO-Porto)/Porto Comprehensive Cancer Center Raquel Seruca (Porto.CCC Raquel Seruca), 4200-072 Porto, Portugal; 3 ICBAS—Institute of Biomedical Sciences Abel Salazar, University of Porto, 4050-313 Porto, Portugal; andre.silva@fc.up.pt; 4 i3S—Institute for Research and Innovation in Health (i3S), University of Porto, 4200-135 Porto, Portugal; 5 INEB—Institute for Biomedical Engineering, University of Porto, 4200-135 Porto, Portugal; 6 REQUIMTE-LAQV, Department of Chemistry and Biochemistry, Faculty of Sciences, University of Porto, 4169-007 Porto, Portugal; 7 Institut Pasteur, Proteomics Core Facility, MSBio UtechS, 75015 Paris, France; rita.pereir@hotmail.com; 8 GlycoMatters Biotech, 4500-162 Espinho, Portugal; 9 Department of Surgical Oncology, Portuguese Oncology Institute of Porto (IPO Porto), 4200-072 Porto, Portugal

**Keywords:** glycomics, glycoproteomics, bladder cancer, targetable biomarkers, GLUT1

## Abstract

Muscle-invasive bladder cancer (MIBC) remains a pressing health concern due to conventional treatment failure and significant molecular heterogeneity, hampering the development of novel targeted therapeutics. In our quest for novel targetable markers, recent glycoproteomics and bioinformatics data have pinpointed (glucose transporter 1) GLUT1 as a potential biomarker due to its increased expression in tumours compared to healthy tissues. This study explores this hypothesis in more detail, with emphasis on GLUT1 glycosylation patterns and cancer specificity. Immunohistochemistry analysis across a diverse set of human bladder tumours representing all disease stages revealed increasing GLUT1 expression with lesion severity, extending to metastasis, while remaining undetectable in healthy urothelium. In line with this, GLUT1 emerged as a marker of reduced overall survival. Revisiting nanoLC-EThcD-MS/MS data targeting immature *O*-glycosylation on muscle-invasive tumours identified GLUT1 as a carrier of short glycosylation associated with invasive disease. Precise glycosite mapping uncovered significant heterogeneity between patient samples, but also common glycopatterns that could provide the molecular basis for targeted solutions. Immature *O*-glycosylation conferred cancer specificity to GLUT1, laying the molecular groundwork for enhanced targeted therapeutics in bladder cancer. Future studies should focus on a comprehensive mapping of GLUT1 glycosites for highly specific cancer-targeted therapy development for bladder cancer.

## 1. Introduction

Muscle-invasive bladder cancer (BLCA) is currently managed by “one size fits all” protocols, where 10–30% of advanced stage patients progress and 50–70% recur after platinum-based neoadjuvant chemotherapy regimen [1,2]. This frequently leads to overtreatment and deleterious side-effects without clear clinical benefits and survival improvements. Recently, immune checkpoint inhibitors (ICI) for BLCA patients were approved, showing modest 20–25% success rates in providing improved overall survival and progression free survival [1,3,4], urging novel therapeutic strategies. Advanced BLCA is characterized by alterations in glycosylation pathways, mainly oversialylation of terminal glycans and consequent arrest in proteins *O*-glycosylation [5]. This event is translated by the overexpression of sialylated short-chain *O*-GalNAc glycans, such as sialyl-Tn (STn) and mono/di sialylated T (ST) antigens [6,7,8,9]. Recently, *O*-glycomics characterization of relevant bladder cancer cell models showing high invasive capacity showed ST antigens as the most abundant cell surface *O*-glycoforms [8]. This was later confirmed in bladder tumours. However, ST antigens were also detected in healthy urothelium, highlighting lack of tumour specificity [8]. To address the need for targetable glycobiomarkers, a more comprehensive interrogation of the cell membrane glycoproteome for biomarker bispecificity was pursued. Glycoproteomics experiments, combining sialidase, lectin affinity, and bottom-up protein identification by nanoLC-ESI-MS/MS in cell lines, identified over 900 glycoproteins carrying ST antigens spanning multiple biological and molecular functions, greatly expanding our view on the bladder cancer glycoproteome (Supplementary Figure S1). Original glycoproteomics data integration with transcriptomics and proteomics information available at public repositories allowed the sorting of glycoproteins that are potentially overexpressed in advanced bladder tumours [8]. An in-house algorithm termed “target score” was then developed to rank glycoproteins according to their potential for targeted therapeutics based on protein expression in healthy and cancerous tissues. Of note, glucose transporter 1 (GLUT1) has emerged as a top-ranked glycoprotein, mostly due to a very restricted expression pattern in healthy tissues and limited off-target effects potential [8]. GLUT1 is the most explored member of the GLUT family, displaying not only elevated affinity for glucose but also for other sugar moieties such as mannose, galactose, and glucosamine [10,11], relevant for glycan biosynthesis. Moreover, several immunohistochemical studies demonstrated that cancer cells typically display increased expression of GLUT1, which correlates with rapid proliferation, enhanced invasion, metastatic potential, unfavourable prognosis, and poor survival in many tumours [12,13,14,15]. In BLCA, GLUT1 sustains the high glycolytic flux of rapidly proliferating cells, playing a key role in the adaptation of bladder cancer cells to microenvironmental challenges [16,17]. However, its exploitation for targeted purposes has been somehow limited by the expression in healthy tissues, supporting deeper investigations for identification of cancer-specific glycoproteoforms [18,19]. This work builds on previous glycoproteomics data [8] and investigates further into GLUT1 expression across different BLCA stages and metastases, emphasizing the clinical relevance of its expression and identifying potentially targetable GLUT1 glycoforms in bladder cancer patients, opening an avenue for targeted intervention in chemotherapy/immunotherapy resistant or refractory patients.

## 2. Results

### 2.1. GLUT1 Glycosylation in Cell Models

**Figure 1:**
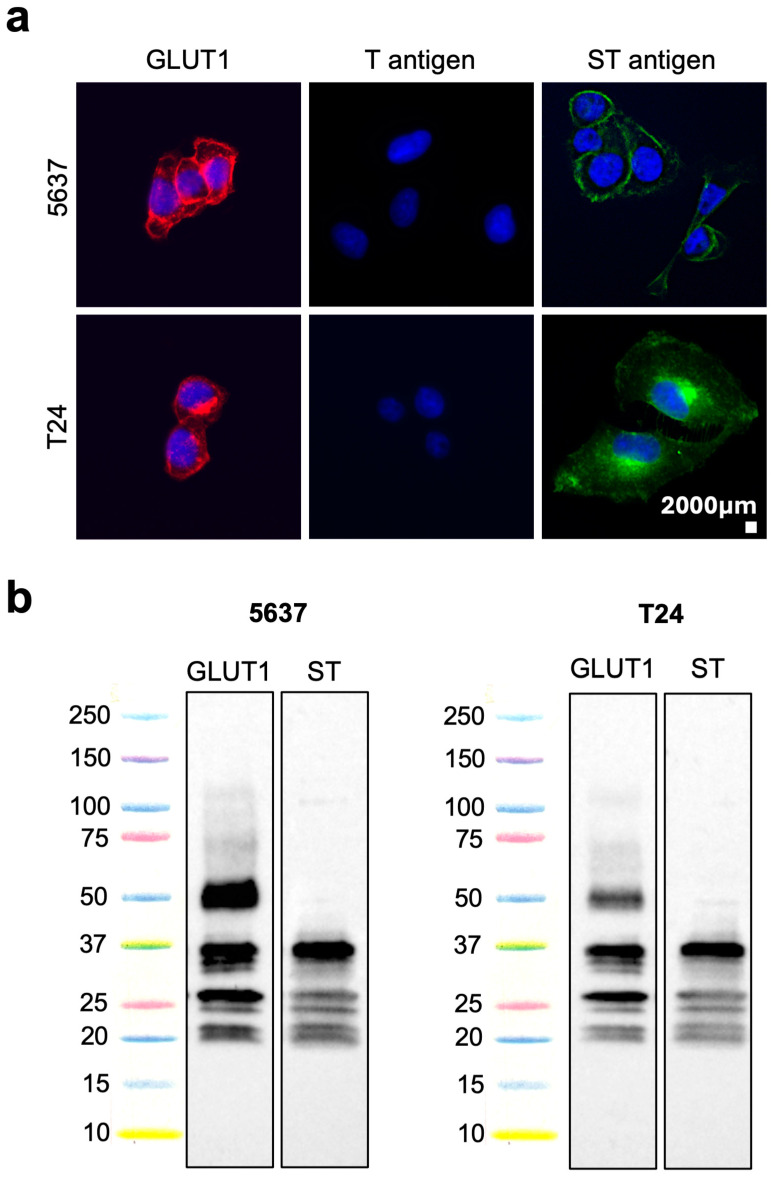
(**a**) Expression of GLUT1 (red), T (green), and ST (green) antigens in 5637 and T24 cell lines. Cells nuclei were stained with DAPI (blue). From left to right, the first panel shows GLUT1 at the cell membrane and, to some extent, in the cytoplasm of both cell lines. The second panel strongly suggests that cell lines did not express the T antigens, while overexpressing ST antigens at the cell membrane as outlined in the third panel. (**b**) Western blot for GLUT1 and ST antigens in GLUT1 immunoprecipitants from 5637 and T24 cell lines. Both 5637 and T24 cell lines demonstrated similar patterns. Namely, the GLUT1 blots show a band above 50 kDa consistent with the molecular weight of canonical GLUT1 forms (55 kDa). It also shows several bands below 37 kDa. The blot for ST antigens supports that lower-molecular-weight isoforms (under 37 kDa) carry ST antigens. Chemiluminescence was detected using a ChemiDoc Imager (Bio-rad, Hercules, CA, USA).

Recent glycoproteomics and bioinformatics data have pinpointed GLUT1 as a potential biomarker due to its increased expression in tumours compared to healthy tissues [8]. Given the potential of GLUT1 for targeted therapeutics, we started by evaluating its cell surface expression by immunocytochemistry (ICC, Figure 1a). GLUT1 was found at the cell membrane and, to some extent, in the cytoplasm of all cell lines. We then addressed GLUT1 *O*-glycosylation to validate glycoproteomics annotations and explore post-translational modifications (PTMs) for improved cancer-specificity. The two cell lines representing different stages of the disease (5637: stage II; T24: stage III) poorly expressed the T antigen by ICC, while being strikingly positive for ST antigens at the cell membrane (Figure 1a). Based on these observations, the more aggressive T24 cell line, expressing both GLUT1 and ST antigens, was used for downstream validation. Briefly, GLUT1 glycoprotein was isolated from enriched membrane protein extracts by immunoprecipitation and analysed by Western blot (Figure 1b). GLUT1 exhibited a main band above 55 kDa, consistent with the canonical form of the glycoprotein, and several other signals below 37 kDa (Figure 1b). According to Uniprot data, these bands may correspond to low-molecular-weight isoforms of this protein. In parallel, immunoprecipitated GLUT1 was also blotted with the Peanut agglutinin (PNA) lectin after neuraminidase treatment to disclose sialylated T antigen expression patterns. Strikingly, only lower-molecular-weight GLUT1 proteoforms were positive for *O*-glycosylated domains, as only faint reactivity was observed for bands at 55 kDa and above (Figure 1b). Taken together, these observations strongly suggest that short-chain *O*-glycosylation is predominantly found in low-molecular-weight GLUT1 proteoforms at the cell membrane.

Given that GLUT1 is a pivotal molecule in regulating responses to microenvironmental stressors, we further investigated how the deficit of glucose and oxygen, often encountered in cancer, impacts the expression of this protein. Accordingly, we subjected 5637 and T24 cells to glucose shortage and acute hypoxia. Both cell lines exhibited similar behaviour, increasing GLUT1 levels in response to low glucose (Supplementary Figure S2), most likely as an attempt to counteract this limitation. This also occurred under low oxygen pressure as well as when both stressors were combined (Supplementary Figure S2). Interestingly, we found that the GLUT1 proteoform at 37 kDa known to carry immunoreactivity to PNA lectin, thereby carrying sialylated T antigens, was particularly affected by changes in the microenvironment.

### 2.2. GLUT1 Expression in Bladder Cancer

**Figure 2:**
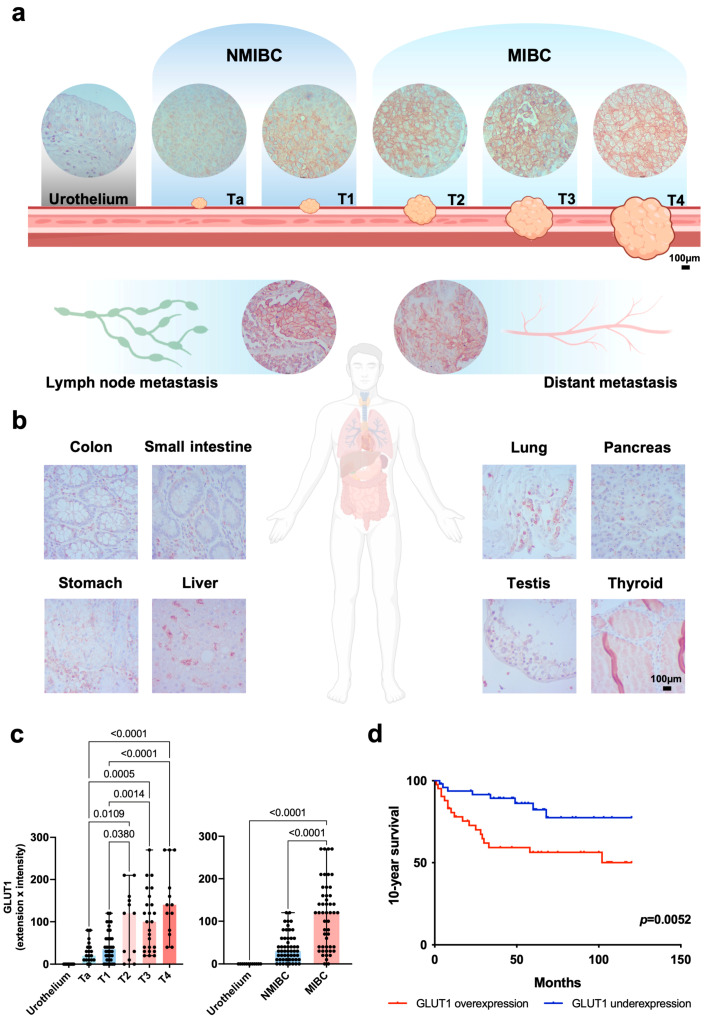
(**a**) Expression of GLUT1 across all stages of BLCA, from healthy urothelium to Ta, T1, T2, T3, and T4 tumours (*n* = 104), lymph node metastases, and distant metastases (*n* = 10). GLUT 1 was not expressed by the healthy urothelium, being increasingly expressed across disease stages and metastases. (**b**) GLUT1 expression in relevant healthy tissues. Thyroid, liver, testis, lung, stomach, pancreas, colon, and small intestine tissue samples were found negative for GLUT1, except for resident leukocyte populations. (**c**) Extension and intensity of GLUT1 expression across disease stages. GLUT1 expression increased with the severity of disease, being overrepresented in muscle-invasive disease compared to non-invasive lesions and the healthy urothelium. Comparisons were performed using an ordinary one-way ANOVA. Statistical significance was considered when *p* < 0.05. (**d**) Ten-year overall survival (OS) of BLCA patients carrying tumours overexpressing GLUT1 vs. patients carrying tumours with GLUT1 underrepresentation. GLUT1 overexpression in BLCA determined decreased 10-year OS of patients. Comparison of estimates was carried out using log-rank tests. Statistical significance was considered when *p* < 0.05.

We then devoted ourselves to a comprehensive characterization of the clinical context of the glycoproteomic signatures previously identified in cell lines, with emphasis on short-chain sialylated *O*-glycans and GLUT1. A series of bladder tumours representative of all stages of the disease, as well as healthy urothelium and metastases, was screened for GLUT1 (Table 1). GLUT1 was not expressed in the healthy urothelium, evidencing its cancer-associated nature. The extension of expression was, in most cases, superior to 20% of the tumour area, predominantly located in apical areas of non-muscle-invasive bladder cancer (NMIBC) and without a clear expression pattern in MIBC (Figure 2a). GLUT1 was detected in the cell membrane and, to a less extent, in the cytoplasm of most primary lesions (94/104) and in all the metastases (Figure 2a and Supplementary Figure S3). Furthermore, tumours with recognizable high density of small-calibre vessels were frequently characterized by low GLUT1 expression. Meanwhile, tumour areas with low density or absence of small-calibre vessels were normally characterized by high GLUT1 and HIF1α expression. Low density of small-calibre vessels is linked with increased nuclear HIF1α expression, and the existence of extensive necrotic areas suggests that GLUT1 expression may be regulated by hypoxic microenvironments in BLCA (Supplementary Figure S3). GLUT1 expression was also addressed in a subset of relevant healthy tissues as thyroid, liver, testis, lung, stomach, pancreas, colon, and small intestine (Figure 2b). Of note, all tissues were negative for GLUT1 except for resident leukocytes populations, which is consistent with The Human Protein Atlas expression annotations. Moreover, the staining intensity and expression levels of GLUT1 increased with the severity of tumour lesions, being more pronounced in latter stages of disease (Figure 2c). In line with this, GLUT1 overexpression was mostly associated with muscle-invasive disease (*p* < 0.0001; Figure 2c). We then evaluated the association of GLUT1 with patient 10-year overall survival (OS). Accordingly, patients with tumours overexpressing GLUT1 presented lower 10-year OS compared to those with low expression (97 vs. 163 months, log-rank *p* = 0.004, Supplementary Table S1). Hence, GLUT1 overexpression constitutes a poor prognostic marker in bladder cancer patients (Hazard Ratio = 1.57; *p* < 0.05, Figure 2d).

### 2.3. GLUT1 Glycosylation in Bladder Cancer

**Figure 3:**
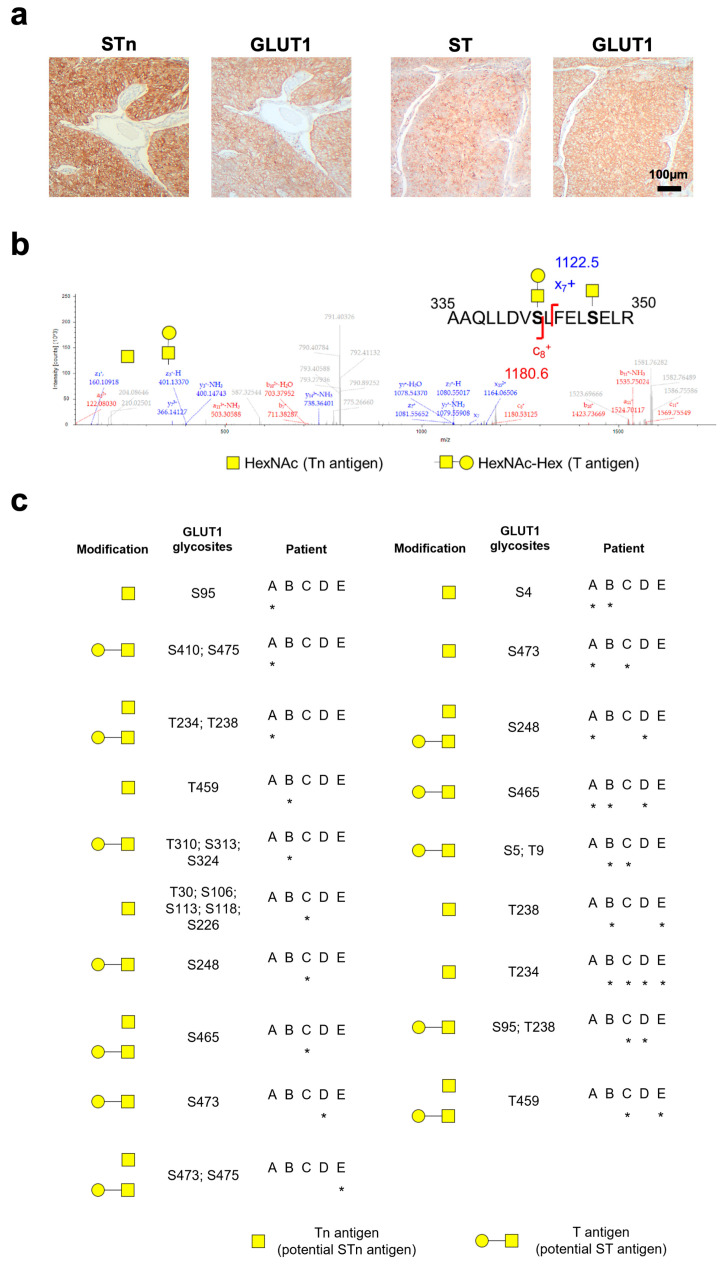
(**a**) Co-localization of STn, ST, and GLUT1 in muscle-invasive BLCA. Co-localization of GLUT1 with STn and ST antigens in the same tumour area suggests that GLUT1 carries these PTMs. (**b**) GLUT1 glycopeptide presenting STn and ST antigens, identified by nanoLC-EThcD-MS/MS. The presence of product ions for HexNAc (Tn; *m*/*z* 204.08) and HexNAcHex (T; *m*/*z* 366.14) modifications (highlighted in the MS/MS spectra) strongly suggests the presence of STn and ST antigens in GLUT1 peptides. Discriminatory fragmentations are also highlighted. (**c**) GLUT1 glycosites identified by nanoLC-EThcD-MS/MS having its canonical isoform as reference. Twenty glycosites were identified for GLUT1, seven of which were observed in two or more samples (S95, T234, T238, S248, S4, S5, T9, T459, S465, and S473), supporting common glycopatterns between patients. STn antigen glycosylation of T234 was found in all samples. * Represents the samples to which the glycosite was assigned.

GLUT1 glycosylation was then assessed envisaging cancer-specific glycosignatures. Five MIBC tumours presenting altered glycosylation translated by the overexpression of both ST and STn antigens were selected for this study. Of note, STn is a cancer-associated short-chain *O*-glycan with a known functional role in BLCA malignancy, characterizing particularly aggressive tumours [6,20]. In all patients, GLUT1-positive tumour areas co-localized with ST and STn (Figure 3a), reinforcing that this glycoprotein could be abnormally glycosylated. As such, a targeted glycoproteomics strategy was adopted to accommodate the presence of both antigens, contemplating first an enrichment for STn-glycoproteins using the VVA lectin after sialidase digestion, followed by a second-dimension affinity chromatography with PNA and nanoLC-EThcD-MS/MS. Glycopeptides presenting the STn (HexNAc as PTM) and ST (HexNAcHex as PTM) antigens or both PTMs were detected in all samples (Figure 3b). Twenty glycosites were identified in GLUT1 (Figure 3c). Moreover, seven GLUT1 glycosites (S95, T234, T238, S4, S5, T9, S248, T459, S465, and S473) were observed in two or more samples, suggesting that some modifications may be conserved across different patients. STn antigen glycosylation of T234 was found in all patient samples (Figure 3c). Moreover, previous data highlighted that the STn antigen is absent from most relevant healthy tissues, apart from mucin secretions of the gastrointestinal tract [8,21]. This data suggests that STn may further improve the cancer specificity of GLUT1, since it has been significantly found in leukocytes, identifying STn-GLUT1 as a BLCA-specific glycosignature.

## 3. Discussion

The current treatment regimen for advanced urothelial carcinoma typically involves platinum-based chemotherapy and immune checkpoint inhibitors. Despite considerable progress in therapeutic approaches, advanced BLCA generally lacks curative options, often exhibiting inherent or acquired resistance to chemotherapy. While immunotherapy presents a more favourable side-effect profile and demonstrates prolonged durable responses compared to traditional chemotherapy, only a minority of patients experience lasting benefits. As such, managing advanced-stage BLCA poses a significant challenge, primarily due to the shortage of clinically approved targeted therapeutics options for locally advanced or metastatic urothelial carcinoma patients who are refractory or resistant to mainstay chemotherapy and immunotherapy [22,23]. This highlights the need for novel cancer-specific biomarkers to foster innovative clinical trials. In pursuit of this objective, our previous research demonstrated that alterations in gene expression and glycome contribute to the remodelling of the membrane glycoproteome in cancer cells [8,24]. These changes often result in cell surface cancer-specific molecular signatures that drive crucial oncogenic processes, while holding substantial potential for clinical interventions. However, studying the membrane glycoproteome remains challenging, requiring the adaptation of conventional proteomics protocols to accommodate structural intricacies arising from glycosylation [25].

Envisaging novel targetable markers, recent glycoproteomics and bioinformatics data has pinpointed GLUT1 as a potential BLCA biomarker due to its increased expression in tumours compared to healthy tissues and its minimal potential for off-target effects [8]. In this work, the clinical relevance of GLUT1 cancer-specific glycoforms was pursued. Herein, GLUT1 was identified at the cell surface of BLCA cells of different molecular and histological backgrounds, reinforcing its pivotal functional role in aggressive cells. Of note, GLUT1 was present not only in its canonical form, but also as low-molecular-weight proteoforms. Interestingly, most of these proteoforms were found carrying short-chain *O*-glycosylation, which reinforces previous findings from our group, and others, emphasizing profound deregulations in glycosylation pathways in bladder cancer [6,26,27,28]. Moreover, less complex glycosylation potentially contributes to decreased GLUT1 molecular weight. We have also attempted to provide the microenvironmental context for low-molecular-weight GLUT1 proteoforms by investigating the impact of oxygen and glucose shortage on BLCA cells, as these are pivotal microenvironmental features of advanced solid tumours. Remarkably, GLUT1 expression was enhanced by glucose shortage and hypoxia, and even more by the combination of both microenvironmental stressors. Moreover, the increased expression of low-molecular-weight GLUT1 proteoforms upon glucose shortage regardless of oxygen tension was evident. This stands in line with our group’s recent findings outlining a general simplification of the tumour cell surface glycome upon glucose and oxygen shortage, mostly due to metabolomic adaptations [29]. Interestingly, these adaptations often culminated in increasing cancer aggressiveness, mostly translated by increased invasion capacity and decreased proliferative potential [29]. Future studies should devote themselves to the investigation of the functional implications of low-molecular-weight GLUT1 glycoforms, envisaging the establishment of the molecular rationale for intervention.

We have also interrogated GLUT1 expression in BLCA tissue samples, focusing on its clinical significance and identification of potentially targetable glycosignatures. Namely, GLUT1 was detected in all stages of BLCA as well as in lymph nodes and distant metastases of primary BLCA tumours.

Of note, tumours characterized by high GLUT1 expression were also characterized by high HIF1α levels, a known transcriptional regulator of glucose transporters, including GLUT1 [30]. Moreover, tumours with elevated GLUT1 expression were normally poorly vascularized with frequently occurring necrotic areas, which are common features of hypoxic tumours. As such, we have investigated the potential link between GLUT1 and HIF1α levels as well as blood vessels density. Of note, low density of small-calibre vessels, along with increased nuclear HIF1α expression and the existence of extensive necrotic areas, suggests that GLUT1 expression may be regulated by hypoxic microenvironments in BLCA. Given the multifactorial regulation of HIF1α in tissues, the molecular mechanisms underlying HIF1α overexpression in BLCA should be addressed in future studies. Furthermore, GLUT1 was progressively more expressed as the disease progressed, being overrepresented in muscle-invasive bladder cancer. Also, GLUT1 was found as a biomarker of poor prognosis in BLCA patients, with impact on decreased 10-year survival. Moreover, GLUT1 was found to be absent from normal urothelium, as well as from a vast panoply of other relevant healthy tissues, except for selected lymphocytic populations. In line with this, we interrogated GLUT1 glycosylation for cancer-specific glycoforms. Building on the *O*-glycomic landscape of bladder tumours, where GLUT1-positive tumour areas co-localized with ST and STn aberrant glycosylation, a guided downstream glycoprotein annotation by mass spectrometry was employed. GLUT1 was found to be glycosylated with the STn and ST antigens in bladder tumours, consistent with observations from cell models. Particularly, the association with STn, rarely observed in healthy organs but significantly overexpressed in more aggressive bladder tumours, enhances the cancer-specific nature of GLUT1-STn glycoforms, also observed in other tumour types [31]. Twenty glycosites on GLUT1 were found to be frequently occupied by short-chain O-glycosylation, translated by STn and ST antigens, in patient samples. Moreover, recurrent GLUT1-STn glycoforms were found in several patients (T234-STn), highlighting common interpatient glycopatterns and paving the way for novel targeted interventions. Based on these preliminary findings, future studies should be devoted to a more comprehensive mapping of GLUT1 glycosites, envisaging the evaluation of aberrantly glycosylated GLUT1 druggability. Upcoming strategies could include a wide panoply of currently used glycan recognizing molecules, such as antibodies, lectins, and other glycan-binding proteins for highly specific cancer-targeted therapeutics. However, there is currently no commercially available solution to address aberrantly glycosylated GLUT1. Several pre-clinical strategies are presently being exploited to precisely target other cancer-associated glycans and glycoproteins within tumour cells, such as monoclonal antibodies [32], antibody-drug conjugates [33], and nanoparticles. Furthermore, the use of nanoparticles in drug delivery presents innovative avenues in cancer treatment, such as vaccines enclosed within synthetic nanoparticles and nanoformulations designed to target glycoproteins or glycan-binding proteins [34,35,36], which could also be envisioned for future GLUT1-targeted therapy. Furthermore, the functional role of clinically relevant GLUT1 glycoforms also warrants future investigation. Notwithstanding, the presented preliminary findings underscore the intricate and dynamic nature of protein glycosylation, emphasizing the importance of understanding glycome structural plasticity in the context of biomarker discovery and therapy development.

## 4. Materials and Methods

### 4.1. Cell Culture

Bladder cancer cell lines reflecting different grades of the disease (5637 and T24) and major bladder carcinogenesis pathways were selected for this study and cultured as described in Peixoto et al. 2021 [8].

### 4.2. Immunocytochemistry

GLUT1 expression was determined in methanol-fixed cells using a rabbit monoclonal [EPR3915] antibody (ab196357, Abcam, Cambridge, UK) incubated 1:100 for 1 h at room temperature. A goat anti-rabbit IgG (H + L) cross-adsorbed secondary antibody Alexa Fluor 594 (ThermoFisher Scientific, A-11012, Waltham, MA, USA) was then incubated for 30 min 1:1000 at room temperature prior to detection. T antigen was detected in PFA 4% fixed cells using 5 μg/mL of fluorescein labelled peanut agglutinin (PNA) lectin (Vector Laboratories, Newark, CA, USA) incubated for 1 h at room temperature. ST antigen detection was performed as described for T antigen after 4 h of cell pellet digestion with 0.1 U/mL of α-neuraminidase (from *Clostridium perfringens* neuraminidase, Sigma, St. Louis, MO, USA) at 37 °C. Immunofluorescence images were acquired using a Zeiss Axio Imager Z1 (Carl Zeiss, Germany) microscope through a Axiocam MR ver3.0 (Carl Zeiss, Germany) camera and using the Software Axiovision 4.8 (Carl Zeiss, Germany).

### 4.3. Immunoprecipitation and Western Blot

A Pierce™ Direct IP Kit (26148, ThermoFisher Scientific, Waltham, MA, USA) was used according to the manufacturer’s instructions to selectively immunoprecipitate GLUT1 from membrane protein lysates of cell lines using a rabbit monoclonal [EPR3915] anti-GLUT1 antibody (1:3000, ab196357, Abcam, Cambridge, UK). The isolated glycoproteins were then run on SDS-PAGE gels, transferred into nitrocellulose membranes, and screened for GLUT1 and ST antigen expression by Western blot using EPR3915 antibody (1:3000) and a biotinylated peanut agglutinin lectin (1:10,000, PNA, B-1075, Vector Laboratories, Newark, CA, USA), respectively. ST antigen detection by PNA lectin was preceded by overnight digestion with 0.1 U/mL α-neuraminidase (from *Clostridium perfringens* neuraminidase, Sigma, St. Louis, MO, USA) in membrane at 37 °C. Horseradish Peroxidase (HRP) goat anti-rabbit secondary antibody (1:60,000; 65-6120, Invitrogen, Waltham, MA, USA) and the Elite ABC Reagent Peroxidase kit (1:10; PK-7100, Vector Laboratories, Newark, CA, USA) were used to detect EPR3915 antibody and PNA lectin, respectively, for 30min at room temperature. Chemiluminescence signals were detected and quantified using a ChemiDoc XRS+ system with Image Lab^TM^ Software 6.0.1 (Bio-Rad, Hercules, CA, USA). Whole protein extracts from 5637 and T24 cell lines under microenvironmental stress (hypoxia and/or glucose deprivation) were also screened for GLUT1 and B2M (1:2500; ab75853, Abcam, Cambridge, UK) by Western blot using the same detection method.

### 4.4. Patient Samples

This study was performed retrospectively in a series of 104 formalin-fixed paraffin-embedded (FFPE) bladder tumours and a panel of 9 heathy tissue samples obtained from archived paraffin blocks at the Portuguese Institute of Oncology—Porto (IPO-Porto), Portugal. Patients were admitted and treated at the IPO-Porto between 2000 and 2015. The series was composed of 84% male and 16% female samples, where patients ranged from 44 to 85 years old (mean age 68 years ± 10 years). The series included 53 non-muscle-invasive bladder tumours, from which approximately 30% were Ta low grade tumours, and the remaining T1 high grade tumours. It also included 51 muscle-invasive bladder tumours representative of all disease stages (12 T2, 25 T3, and 14 T4) and 10 metastases (5 lymph node; 5 distant metastases). The time of follow-up was on average 49 months (1–226 months). Ten-year overall survival (OS) was defined as the period between surgery and the last follow-up evaluation in the subsequent ten years or the occurrence of death by cancer. All procedures were performed under patients’ informed consent and after approval by the IPO-Porto ethics committee. Clinicopathological information was obtained from patients’ clinical records. Healthy urothelium, thyroid, liver, testis, lung, stomach, pancreas, small intestine, and colon tissue sections were also included in the comparative study.

**Table 1:** Clinicopathological variables associated with the employed retrospective BLCA series.

	n (%)
Non-muscle-invasive	53 (50.9)
Muscle-invasive	51 (49.1)
Stage	
Ta low grade	19 (18.3)
T1 high grade	34 (32.7)
T2	12 (11.5)
T3	25 (24.0)
T4	14 (13.5)
Lymph node metastasis (N)	
No	13 (12.5)
Yes	15 (14.4)
Missing information	76 (73.1)
Distant metastasis (M)	
M0	48 (46.2)
M1	2 (1.9)
Missing information	54 (51.9)

### 4.5. Bladder Tumours Glycoproteomics

Five MIBC showing high STn and ST expressions by immunohistochemistry were selected for tumour glycoproteomics. Proteins were extracted from formalin-fixed paraffin-embedded tumour tissues using the Qproteome FFPE tissue kit (QIAGEN, Hilden, Germany) according to the vendor’s instructions. Protein extracts were submitted to glycoproteomics analysis as described in Peixoto et al. 2021 [8].

### 4.6. Tissue Expressions of GLUT1, STn and ST Antigens

Formalin-fixed paraffin-embedded (FFPE) tissue sections were screened by immunohistochemistry for GLUT1 and ST antigens using the streptavidin/biotin peroxidase method as described previously [6] using the above-mentioned antibody (1:500, 30 min. room temperature) and PNA lectin (α-neuraminidase digestion at 37 °C, following 1 h incubation with 100 ug/mL PNA). STn detection was achieved using an Anti-tag-72 antibody [B72.3 + CC49] (ab199002, Abcam, Cambridge, UK) 0.5 μg/mL overnight at 4 °C and HIF1α was detected using an anti-HIF1α (ab51608; Abcam, Cambridge, UK) at 1:600 for 30 min at room temperature. Immuno-reactive sections were blindly assessed using light microscopy by two independent observers and validated by an experienced pathologist. Briefly, ST and GLUT1 were scored according to a semi-quantitative approach based on the intensity and extension of the staining. The extension of staining was rated in cutoffs of 10%, and staining intensity was rated as follows: negative—0, weak—1, moderate—2, and strong—3 points. The tumours were then classified based on the multiplication of extension evaluation and intensity. Non-consensual readings were re-analysed using a double-headed microscope and consensus was reached. For STn and HIF1α classification, cellular location (STn: cytoplasm, plasma membrane; HIF1α–nucleus) and positivity were considered whenever the antigen was present.

### 4.7. Statistical Analysis

Statistical analysis was performed using Statistical Package for Social Sciences–SPSS for MacOS (version 27; IBM) and GraphPad Prism software (version 9; Dotmatics, Bishop’s Stortford, UK). Chi-square analysis was used to compare categorical variables and an ordinary one-way ANOVA and unpaired *t* test with Welch’s correction were used for continuous variables. Kaplan–Meier survival curves were applied to evaluate correlation between the evaluated biomarker and the 10-year overall survival (OS). Comparison of estimates was carried out using log-rank tests.

## 5. Conclusions

This study provides a detailed exploration of GLUT1 as a potential biomarker for muscle-invasive bladder cancer (MIBC), elucidating GLUT1’s significance in disease progression and prognosis. Immunohistochemistry analysis revealed a consistent increase in GLUT1 expression with advancing disease stages, including metastases, while remaining absent in healthy urothelium. Moreover, GLUT1 emerged as a marker associated with reduced patient survival, emphasizing its clinical relevance. Notably, immature O-glycosylation, translated by STn expression, conferred cancer specificity to GLUT1, circumscribing GLUT1-STn expression to tumour cells. NanoLC-EThcD-MS/MS data further identified GLUT1 as a putative carrier of short-chain glycosylation associated with aggressive forms of the disease, as the STn antigen. The precise mapping of glycosites uncovered both interpatient heterogeneities and common glyco-patterns. Namely, STn antigen glycosylation of T234 was found in all patient samples, offering crucial insights for targeted therapeutic interventions. Moving forward, future studies should prioritize comprehensive mapping of GLUT1 glycosites to facilitate the development of highly specific cancer-targeted therapies for MIBC.

## Data Availability

Data is contained within the article and Supplementary Material.

## Supplementary Materials

The supporting information can be downloaded at: https://www.mdpi.com/article/10.3390/ijms25063462/s1.
