# Supplementary material for: Aberrantly Glycosylated GLUT1 as a Poor Prognosis Marker in Aggressive Bladder Cancer
Source: Int J Mol Sci. 2024 Mar 19;25(6):3462. doi: 10.3390/ijms25063462 (PMC10971045; doi:10.3390/ijms25063462)
Supplement: Supplementary file 1 [file ijms-25-03462-s001.zip › ijms-2892909-supplementary.pdf]

Communication

Aberrantly Glycosylated GLUT1 as a Poor Prognosis marker in Aggressive Bladder Cancer

Eduardo Ferreira[1,2], Dylan Ferreira[1,2,3,4], Marta Relvas-Santos[1,2,3,4,5], Rui Freitas[1,2,3,4], Janine Soares[1,2,3], Rita Azevedo[6], Luís Pedro Afonso[1,2,] Luís Lima[1,2], Beatriz Santos[1,2], Martina Gonçalves[1,2], André M. N. Silva[3,5,7] Lúcio Lara Santos[1,2,7,8], Andreia Peixoto[1,2,4*] and José Alexandre Ferreira[1,2,3,7*]

1. Experimental Pathology and Therapeutics Group, Research Center of IPO-Porto (CI-IPOP), 4200-072 Porto, Portugal;
2. RISE@CI-IPOP (Health Research Network), Portuguese Oncology Institute of Porto (IPO-Porto) / Porto Comprehensive Cancer Center Raquel Seruca (Porto.CCC Raquel Seruca), 4200-072 Porto, Portugal;
3. ICBAS - Institute of Biomedical Sciences Abel Salazar, University of Porto, 4050-313 Porto, Portugal;
4. i3S - Institute for Research and Innovation in Health (i3S), University of Porto, 4200-135 Porto, Portugal / INEB - Institute for Biomedical Engineering, University of Porto, 4200-135 Porto, Portugal;
5. REQUIMTE-LAQV, Department of Chemistry and Biochemistry, Faculty of Sciences, University of Porto, 4169-007 Porto, Portugal;
6. Institut Pasteur, Proteomics Core Facility, MSBio UtechS, 75015 Paris, France;
7. GlycoMatters Biotech, 4500-162 Espinho, Portugal;
8. Department of Surgical Oncology, Portuguese Oncology Institute of Porto (IPO Porto), 4200-072 Porto, Portugal

* Correspondence: AP, andreia.peixoto@ipoporto.min-saude.pt, Tel. +351 225084000 (ext. 5111); JAF, jose.a.ferreira@ipoporto.min-saude.pt, Tel. +351 225084000 (ext. 5111);.

--Supplementary Information--

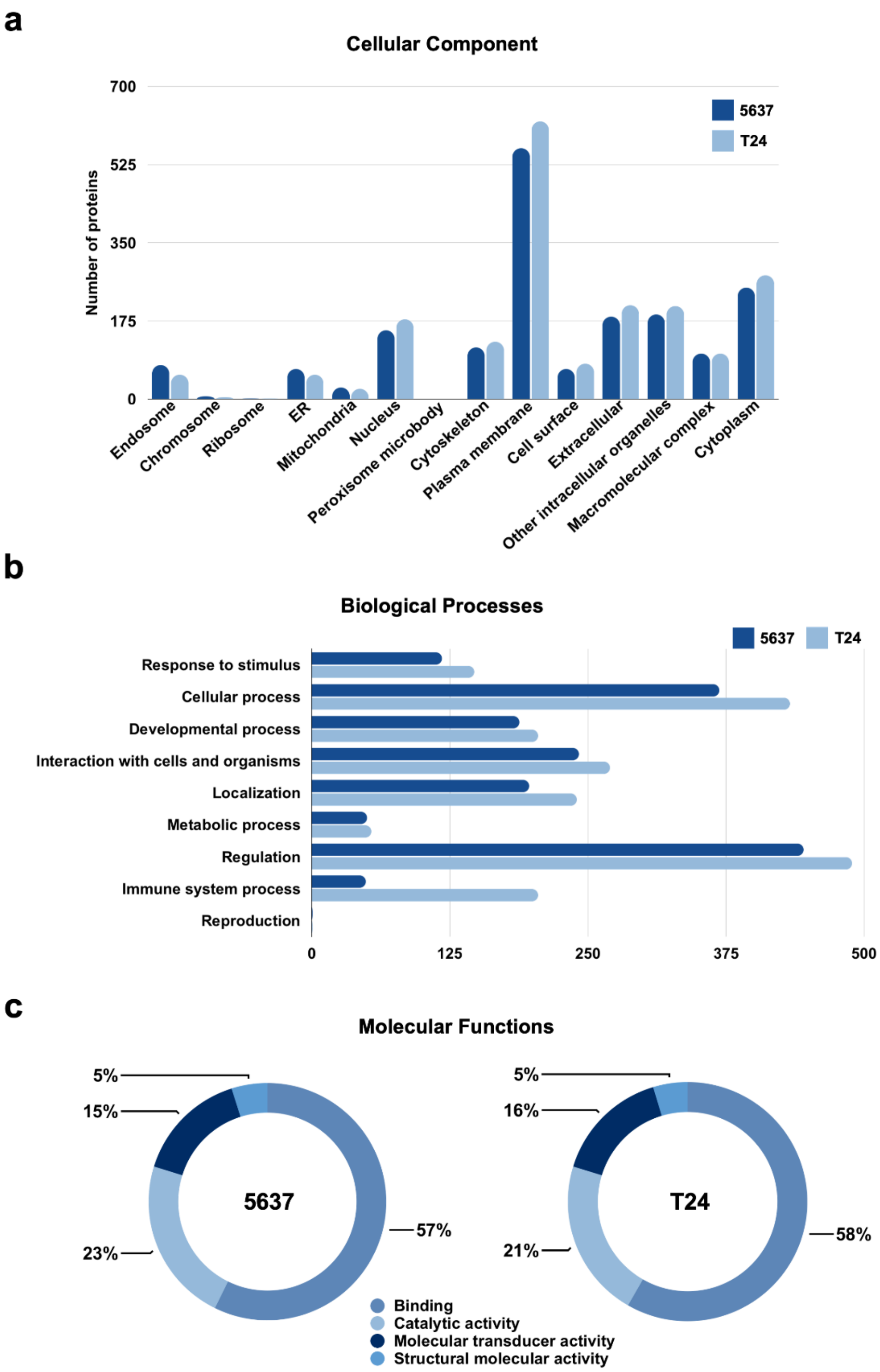

Supplementary Figure S1. a) Sub-cellular location, b) biological processes and c) molecular functions associated to the identified glycoproteins. 900 glycoproteins were comprehensively analyzed based on GO terms for sub-cellular location, biological processes, and molecular functions. Chart A shows that identified glycoproteins are not restricted to the plasma membrane and extracellular domains and can also be found in the cytoplasm, cytoskeleton, nucleus, and other locations, suggesting a broad range of molecular and

functional roles. These glycoproteins are involved in wide arrays of biological functions including cell regulation, immune system mediation, interactions with cells and organisms, response to stimuli and many other cellular processes. This was confirmed by subsequent bioinformatics analysis, showing as main molecular functions binding, catalytic and molecular transducer activities.

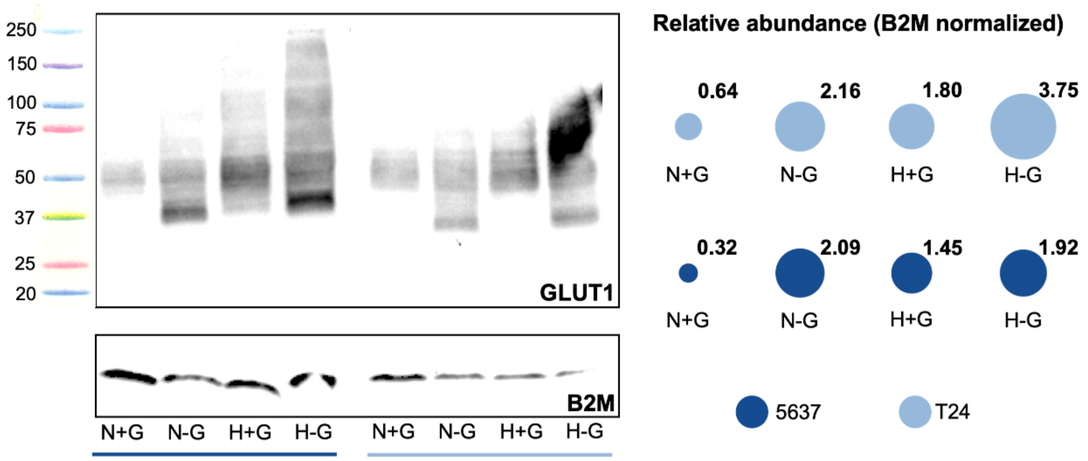

Supplementary Figure S2. 5637 and T24 cells were subjected to hypoxia (H+G), glucose shortage (N-G) and both hypoxia and restriction of glucose (H-G). Both cell lines exhibited similar behavior, increasing GLUT1 levels in response to low glucose and under low oxygen pressure as well as when both stressors were combined. The proteoform at 37 kDa found to carry sialylated T antigens was found to be overrepresented upon the induction of glucose shortage. Chemiluminescence signals were detected through a ChemiDoc imager. The estimated quantification refers to the pixel density in every lane. Results correspond to the mean value of three independent experiments.

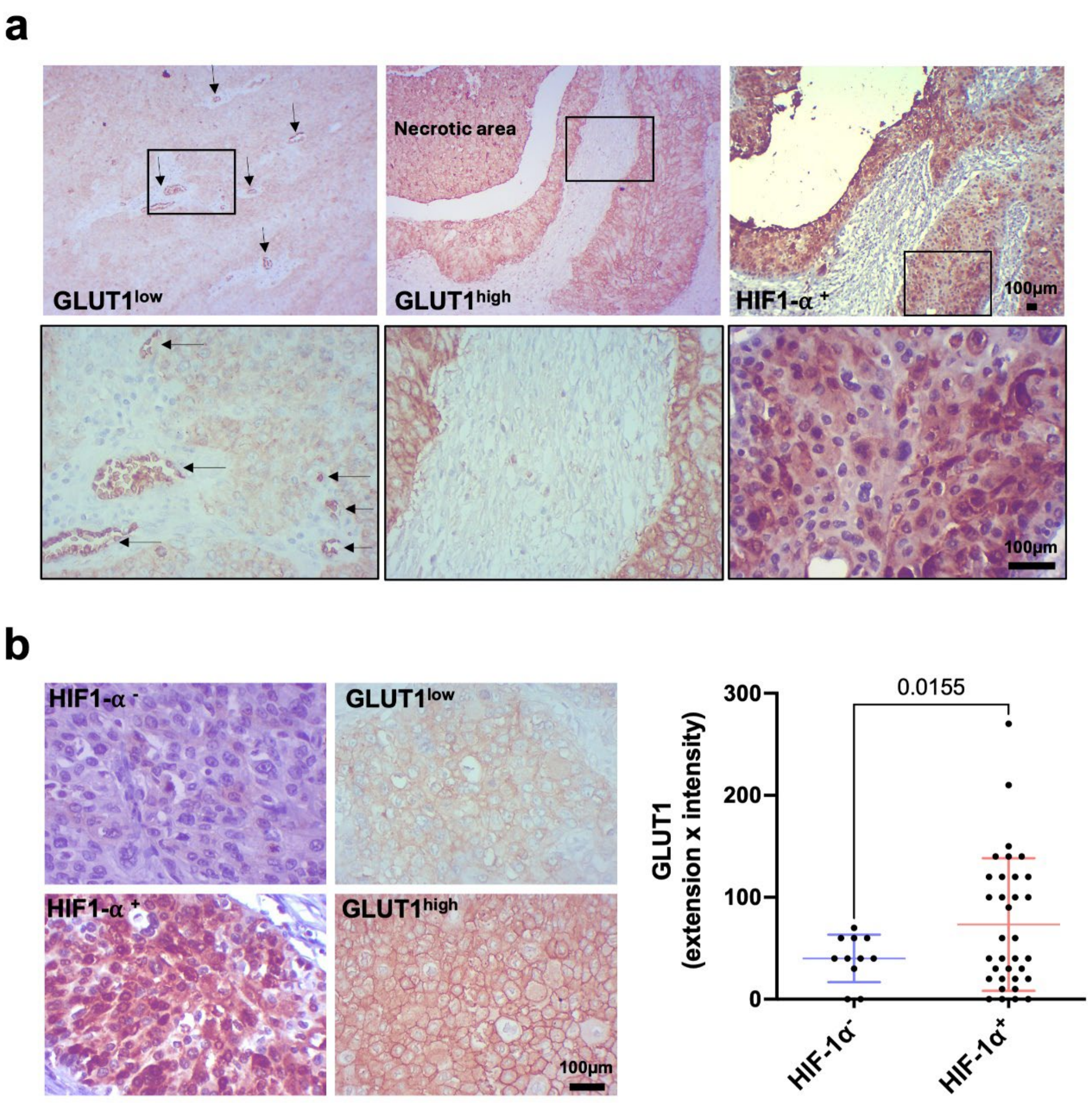

Supplementary Figure S3. a) Micrographs of BLCA tumours representative of the association between blood vessels density and overall GLUT1 expression. Tumours with recognizable high density of small caliber vessels (arrows) are frequently characterized by low GLUT1 expression. Meanwhile, tumour areas with low density or absence of small caliber vessels are normally characterized by high GLUT1 and HIF1α expression (right side panels). Low density of small caliber vessels, allied to increase nuclear HIF1α expression and the existence of extensive necrotic areas suggests that GLUT1 expression may be regulated by hypoxic microenvironments in BLCA tumours. **b)** Analysis of GLUT1 expression in HIF1α + tumours compared to HIF1α^{-} tumours. Non-consecutive sections were used for the analysis. Of note, GLUT1 membrane expression is evidenced. The graph represents the mean extension x intensity of 46 BLCA cases plus standard deviation. Namely, HIF1α^{-} tumours displayed a mean extension x intensity of GLUT1 of 73 ±65, while HIF1α^{+} tumours displayed a mean extension x intensity of GLUT1 of 40 ±23. This highlight that potentially hypoxic tumours overexpressing the hypoxia marker HIF1α display increasing amounts of GLUT1, in what appears to be an adaptation to the microenvironmental challenge. Comparisons were performed using an unpaired t-test with Welch's correction, after normality evaluation (Shapiro-Wilk). Statistical significance was considered when $p<0.05$.

Supplementary Table S1. Clinicopathological variables comparison between patients bearing tumours with low GLUT1 expression vs. GLUT1 overexpression.

	**GLUT1 low expression**	**GLUT1 overexpression**	
	n (%)	*n* (%)	*p* value
Non muscle invasive	8 (80.0)	45 (47.9)	0.093
Muscle invasive	2 (20.0)	49 (52.1)	
**Non muscle invasive**			
Ta low grade	2 (25.0)	17 (37.8)	0.395
T1 high grade	6 (75.0)	28 (62.2)	
**Muscle invasive**			
T2	2 (100.0)	10 (20.8)	-
T3	0 (0)	25 (52.1)	0.099
T4	0 (0)	13 (27.1)	0.220
**Lymph node metastasis (N)**			
No	1 (100.0)	12 (44.4)	0.464
Yes	0 (0)	15 (55.6)	
**Distant metastasis (M)**			
M0	2 (100.0)	46 (95.8)	0.921
M1	0 (0)	2 (4.2)	
